# Novel *LRF*/*ZBTB7A* variants and known HbF-modulating SNPs in transfusion-dependent β-thalassemia

**DOI:** 10.1186/s12920-025-02275-5

**Published:** 2025-12-18

**Authors:** Yunus Arikan, Tugba Karaman Mercan, Merve Embel, Erdal Kurtoglu

**Affiliations:** 1 Medicine Faculty, Department of Medical Genetics, Yozgat Bozok University, Yozgat, Turkey; 2 Medicine Faculty, Department of Medical Biology and Genetics, Akdeniz University, Antalya, Turkey; 3 Department of Internal Medicine, Division of Hematology, Antalya Training and Research Hospital, Antalya, Turkey

**Keywords:** ZBTB7A/LRF, Fetal hemoglobin, Beta thalassemia, K562, Bioinformatics

## Abstract

**Background:**

β-Thalassemia is a hereditary blood disorder with a highly variable clinical presentation that is partly influenced by genetic modifiers that regulate fetal hemoglobin levels. Elevated HbF can ameliorate the clinical symptoms of β-thalassemia, making the identification of HbF-modifying loci crucial for understanding disease variability and developing potential therapeutic strategies.

**Methods:**

In this study, we evaluated the distribution and effect of known HbF-associated single nucleotide polymorphisms within the *BCL11A*, *HMIP*, and *XmnI-HBG2* loci in β-thalassemia patients. Our investigation was initiated by a hypothesis based on genomic data from the K562 cell line, which indicated the presence of a specific *LRF*/*ZBTB7A* variant. In addition, we explored the role of *LRF*/*ZBTB7A*, a transcription factor implicated in globin gene regulation, through Sanger sequencing and in silico analyses. A hypothetical Genetic Modifier Score was devised to quantify the cumulative effect of the five major HbF-associated QTLs.

**Results:**

While we confirmed the previously reported associations of the common SNPs with HbF levels, we also identified novel rare variants in *LRF*/*ZBTB7A* (p.Pro241Leu, p.Asp344Asp, p.Glu277del) that may influence HbF expression. *XmnI-HBG2* accounted for a small yet significant proportion (7%) of HbF variability. A significant positive correlation was found between the Genetic Modifier Score and HbF levels, yet the model explained only ~ 14% of the variance, highlighting a substantial role for other modifiers such as the novel *LRF*/*ZBTB7A* variants identified here.

**Conclusions:**

These results suggest *LRF/ZBTB7A* is a novel modifier of HbF. The discovery of the variants, against a backdrop of modest QTL effects, underscores its potential and warrants further functional investigation.

**Supplementary Information:**

The online version contains supplementary material available at 10.1186/s12920-025-02275-5.

## Background

Beta thalassemia (OMIM #613985) is the most common hereditary blood disease and is a Mendelian disease with a well-known genotype‒phenotype correlation and autosomal recessive inheritance [1]. Homozygous or compound heterozygous mutations in the beta globin (*HBB*) gene (*141900) cause beta thalassemia. The amount of fetal hemoglobin (α2γ2) is among the genetic factors that affect the clinical outcome of this disease [2]. Increasing the amount of fetal hemoglobin is one of the strategies that has been implemented in beta-hemoglobinopathies [3, 4].

Fetal hemoglobin induction via lentiviral gene therapy and CRISPR-based clinical trials (NCT03655678, NCT03745287, and NCT05477563) are promising for the treatment of beta thalassemia or sickle cell disease [5, 6].

The modifier genes that endogenously determine the fetal hemoglobin level (quantitative loci for the hereditary persistence of fetal hemoglobin (QTL)) are the *HBG2*-*XmnI* (rs7482144) polymorphism, the intergenic variants in the *HBS1L-MYB* (*HMIP*) locus, and the single nucleotide polymorphisms in the *BCL11A* (*142335) gene [7]. The *LRF*/*ZBTB7A* (*605878) gene, which is impaired in the pathogenesis of acute myeloid leukemia, was found to be an independent repressor of the fetal hemoglobin induction pathway at the same time [8, 9].

Despite advances in understanding HbF regulation, interindividual variation in response to known modifiers suggests the presence of additional genetic factors. In the present study, we genotyped 2 SNPs in the *BCL11A* gene together with 2 SNPs in the *HMIP* region and the *XmnI*-*HBG2* polymorphism via the reverse dot blot hybridization-based method (RDBH) in 58 patients with TDBT. We first showed how much the 5 most frequently studied SNPs could increase the amount of HbF in our beta thalassemia cohort. Later, as a result of bioinformatics screening for the detection of specific candidate variation (*LRF/ZBTB7A*, p.P241L), we revealed novel mutations of a candidate gene that could affect only the amount of HbF for the first time in our cohort.

## Methods

### Study design and ethical approval

The local ethical committee approved the current prospective study (KAEK-189_2019.03.13_12). Written informed consent was given by all participants in accordance with the Declaration of Helsinki. DNA isolation (QIAAmp^®^ DNA extraction blood minikit, Qiagen) was performed from the peripheral blood of 58 TDBT patients and 60 controls who signed informed consent forms. The HbF levels of patients (HbF > 2%) and controls (HbF ≤ 2) were determined via the Bio-Rad Variant™ Hemoglobin Testing System II.

### Genotyping strategy

Comprehensive *HBB* genotyping to confirm the beta-thalassemia diagnosis was performed only for the TDBT patients, using data retrieved from their clinical archives. For the control group, *HBB* genotyping was not clinically indicated; their inclusion served to establish a population baseline for *LRF/ZBTB7A* variant screening and HbF distribution.

Genotyping for 5 SNPs (rs7482144, rs1427407, rs10189857, rs28384513, and rs9399137) was performed via the RDBH-based strip method, ViennaLab StripAssays^®^ (ViennaLab Diagnostics GmbH). This assay is based on the use of subsequent multiplex PCR and RDBH. To best reflect endogenous production, HbF levels were measured from blood samples obtained just before a scheduled transfusion.

### Targeted Sanger sequencing based on cell line hypothesis

Our investigation was initiated by a hypothesis based on prior genomic data from the K562 cell line, which indicated the presence of the specific *LRF/ZBTB7A* p.P241L (c.722 C > T) variant. To test the clinical relevance of this observation, we designed a targeted Sanger sequencing approach.

Genomic DNA from 58 TDBT patients and 60 controls was used to amplify the specific region of exon 2 of the *LRF/ZBTB7A* gene (NC_000019.9) harboring the c.722C > T locus using the following primers: forward, 5’-ATGACCTGGATGCCACCAAG-3’; reverse, 5’-GCCTTGGCTCGGATCTTCTT-3’. The resulting PCR products were sequenced on an ABI-3130-XL Genetic Analyzer (Applied Biosystems).

The possible damaging effects of the identified mutations were evaluated by considering the American College of Medical Genetics and Genomics (ACMG) guidelines and Mutation Taster database. In silico analyses for splice prediction (SpliceAI), evolutionary conservation (PhyloP), codon usage (https://www.bioinformatics.org/sms2/codon_usage.html), and pathogenicity (CADD, AlphaMissense) were also performed. Variant-specific ACMG pathogenicity criteria, gnomAD allele frequencies, and population-specific allele frequencies for Turkiye for the *ZBTB7A* gene variations were individually retrieved from the https://search.ngscloud.com/portal.

### Data analysis

Nonparametric Mann‒Whitney U and Kruskal‒Wallis tests were performed via SPSS version 20 (IBM SPSS Statistics for Windows-MacOS) to assess the effects of relevant SNPs on HbF levels. The extent to which SNPs could explain the HbF level was determined via univariate analysis. Linkage disequilibrium (LD) scores of two SNPs of each *BCL11A* and *HBS1L-MYB* were retrieved from the database for all human populations (https://ldlink.nih.gov/?tab=ldpop). The genotypic and allelic frequencies of the SNPs in our patient group were also calculated.

### Genetic modfifier score calculation

To quantitatively assess the cumulative contribution of known Hb F-associated quantitative trait loci (QTLs), a hypothetical Genetic Modifier Score was devised. The score was based on five major QTLs (specify loci if desired, e.g., *BCL11A*, *HBS1L-MYB*, and *XmnI-HBG2*). For each SNP, genotypes were assigned a value of 0, 1, or 2 points based on established literature, corresponding to the number of HbF-increasing alleles. The individual SNP scores were then summed to create a cumulative Genetic Modifier Score for each patient, where a higher score indicates a greater predicted genetic predisposition for elevated HbF production. A patient with a genetic modifier score of “2-1-0-0-0” would receive a total of 3 points (calculated as 2 + 1 + 0 + 0 + 0). The theoretical maximum of the genetic risk score is 10 points.

## Results

**Fig. 1:**
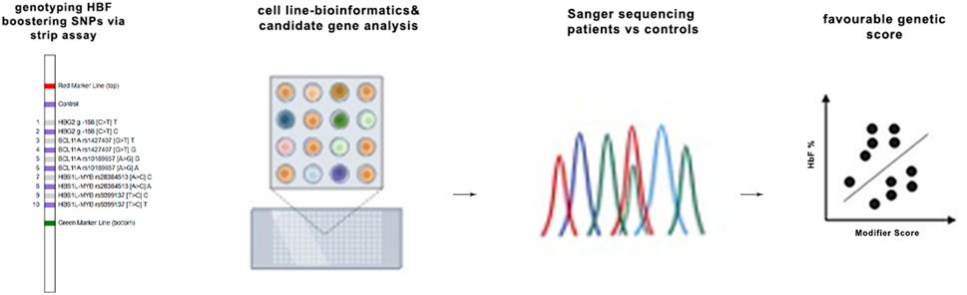
Graphical abstract summarizing the study design and concept. First, known QTLs that cause high HbF levels were genotyped in only TDBT patients via the RDBH assay. Second, regarding the literature and obtaining a deep genomic profile of the K562 cell line, a certain mutation in the exon 2 region of *LRF*/*ZBTB7A* was selected, and this mutation was screened in both patients (n=58) and controls (n=60) via Sanger sequencing. A favorable genetic modifier score was calculated for each patient to predict disease severity and clinical outcomes

The study followed a three-step design, including QTL genotyping, candidate mutation selection in *LRF/ZBTB7A*, and screening in both patients and controls, as shown in Fig. 1. The mean age of the patients was 32.4 ± 9.3 years, and the mean HbF level was 8.8 ± 5.6%, with values ranging from 2.0% to 24.4%. The presence of the T allele of *XmnI-HBG2* was significantly associated with higher HbF levels (*p* = 0.032, Mann‒Whitney U test), even though the overall genotype effect on HbF was not statistically significant (*p* > 0.05, Kruskal‒Wallis tests). There was also a significant difference between being above the mean HbF value of 8.8 and having the T allele (*p* = 0.046, Fisher’s exact test). Furthermore, we showed that the *XmnI-HBG2* polymorphism explained 7.0% of the variance in HbF levels after adjusting for age (ANCOVA, F(2,54) = 4.093, *p* = 0.048; *n* = 57 due to one missing age value), marking the first such report in the Turkiye population (Supplementary Table S1).

**Fig. 2:**
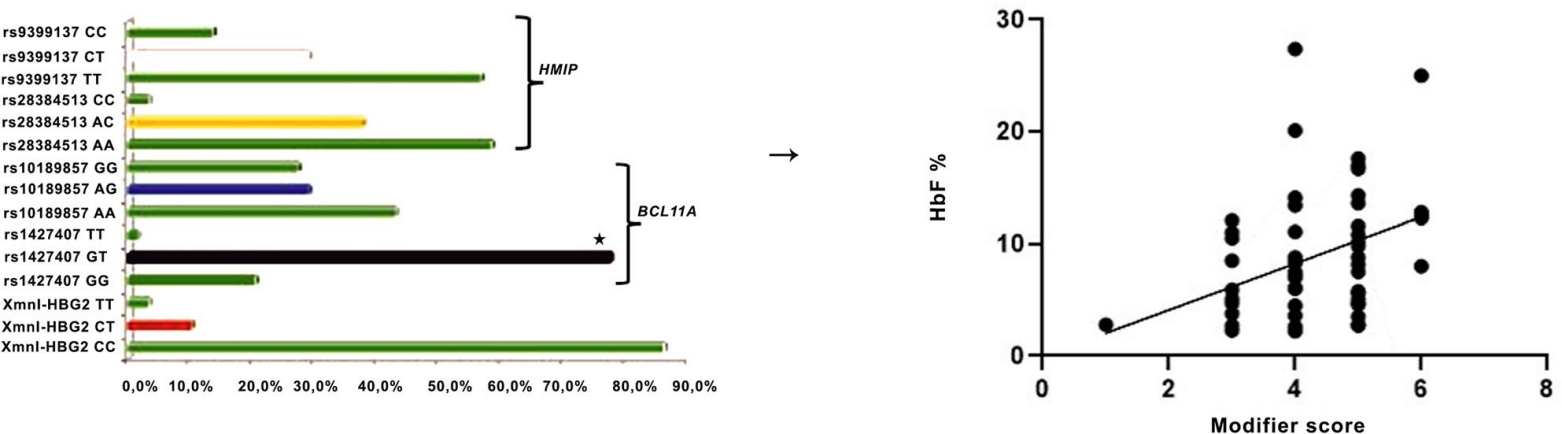
The relationship between the cumulative genetic modifier score and HbF levels. The *BCL11A* rs1427407 GT genotype presented the highest heterozygosity rate among the key HbF modulating loci in the TDBT cohort. On the right panel, the score calculated from five major HbF associated loci, demonstrated a significant positive correlation with HbF%, underscoring the collective contribution of these genetic modifiers to fetal hemoglobin variation in the cohort

No relationship was found between the presence of a certain allele of the other 4 SNPs and the HbF level. In terms of genotype frequencies (Fig. 2), the SNP with the highest heterozygosity was *BCL11A* rs1427407 (77.6%), whereas the lowest was *XmnI*-*HBG2* rs7482144 (10.3%). The LD scores of the SNPs (rs1427407 and rs10189857) in *BCL11A* were 0.25, and the LD scores were 0.13 for the SNPs (rs28384513 and rs9399137) in *HBS1L-MYB* (Table 1). When the minor allele frequency of SNPs in the normal population was compared with the allele frequencies in our patient population, the greatest difference was detected in rs9399137 in *HBS1L-MYB.*

**Table 1:** Baseline characteristics and frequencies of HbF-modifying variants

Modifier	Genotype (%)	%HbF (median)	*P* value*	MAF^a^	Allele Freq.^b^	LD (*r*^2^)
*XmnI-HBG2* rs7482144	CC (86.2)	C allele (7.5)		0.74	0.91	
	CT (10.3)	T allele (10.6)	0.032	0.26	0.09	
	TT (3.5)					
*BCL11A* rs1427407	GG (20.7)	G allele (7.5)		0.80	0.54	
	GT (77.6)	T allele (6.8)	0.328	0.20	0.46	
	TT (1.7)					0.25
*BCL11A* rs10189857	AA (43.1)	A allele (8.1)		0.49	0.58	
	AG (29.3)	G allele (7.2)	0.195	0.51	0.42	
	GG (27.6)					
*HBS1L-MYB* rs28384513	AA (58.6)	A allele (8.1)		0.74	0.78	
	AC (37.9)	C allele (8.4)	0.497	0.26	0.22	
	CC (3.4)					0.13
*HBS1L-MYB* rs9399137	TT (56.9)	T allele (8.8)		0.85	0.28	
	TC (29.3)	C allele (8.1)	0.187	0.15	0.72	
	CC (13.8)					

*LD* Linkage disequilibrium score (r²) retrieved for all populations from the LDlink database

*Mann-Whitney U test

^a^The Minor Allele Frequency reported for the general population in the 1000 Genomes Project

^b^The frequency of each allele calculated from the genotype data within our study cohort

**Fig. 3:**
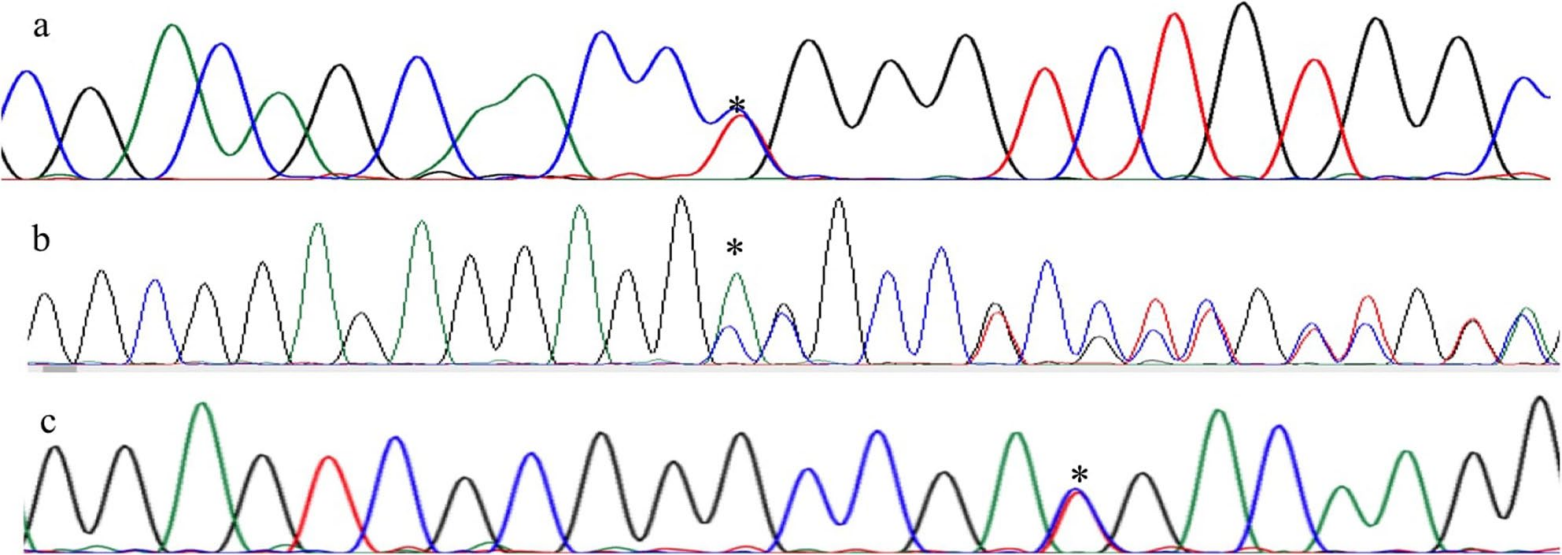
Representative Sanger sequencing chromatograms of *LRF*/*ZBTB7A* variants observed in the study. **a** Heterozygous rs538445777 (c.722C>T) in a patient with elevated HbF (3.5%); **b**) In-frame deletion rs1361497779 (c.829_831delGAG) in a control subject (HbF: 1.5%); **c**) rs112339723 (c.1032C>T) in a patient with an HbF level of 5.1%

In the *LRF/ZBTB7A* gene, the investigation was motivated by an initial screening of an archived version of the DepMap database, which contained an entry for the p.P241L variant in the K562 cell line. To assess the clinical relevance of this observation, we performed targeted Sanger sequencing in our cohort. This analysis confirmed the presence of the p.P241L variant (rs538445777, c.722 C > T) in a patient with an HbF value of 3.5% (Fig. 3a). Additionally, a novel in-frame deletion (c.829_831del, p.E277del, rs1361497779) was detected in a control group individual with an HbF value of 1.5% (Fig. 3b), and a synonymous mutation (rs112339723, p.D344D) was found in one patient with an HbF value of 5.1% (Fig. 3c). The rs538445777, rs1361497779 and rs112339723 variations in the *LRF/ZBTB7A* gene were evaluated as VUSs according to the ACMG criteria (Table 2).

**Table 2:** Genetic and hematological profiles of cases with *ZBTB7A* variants

Variation	Protein	% MAF (gnomAD)	% in Turkiye	ACMG criteria	^a^SNP profile	% HbF	% HbA2	HGB (g/dL)	MCV (fL)	HBB^b^
rs538445777	p.P241L	0.05	0.19	PM1	1-1-0-0-0	3.5	2.8	9.5	85.4	−30 T > A/IVS.I.110 (G > A)
rs1361497779	p.E277del	**-**	**-**	PM2	-	1.5	-	-	-	-
rs112339723	p.D344D	0.14	0.31	BS2	1–2-0-0-0	5.1	2.4	8.2	77.9	IVS.I.110 (G > A)/IVS.I.110 (G > A)

0:homozygous normal, 1:heterozygous, 2 homozygous mutated allele. First two SNPs are located in *BCL11A* gene, following two SNPs are located in *HBS1L-MYB* locus. The last SNP (rs7482144) is also known as *XmnI-HBG2*

^a^The five HbF modifier SNPs are rs1427407-rs10189857-rs2838451-rs9399137-rs7482144, respectively

^b^The complete dataset for all study participants is available in Supplementary Table S2. p.P241L and p.D344D (TDBT patients); p.E277del (control)

In the archive records (Supplementary Table S2), the *HBB* gene mutation in the patient whose HbF value was 3.5 was − 30 T > A/IVS.I.110 (G > A). The patient was splenectomized and received a blood transfusion once a month. This patient has the p.P241L mutation in the *LRF/ZBTB7A* gene, and the HbF modifier SNP profile is 1-1-0-0-0. Although the *HBB* profile of the individual with an HbF value of 1.5 in the control group is unknown, the only difference in the HbF modifier profile is the homozygous mutation of the rs10189857 SNP in *BCL11A* (Table 2). The patient was splenectomized and had a homozygous IVSI-110 (G > A) mutation in the *HBB* gene with a recorded HbF level of 5.1%.

To quantitatively assess the cumulative effect of known HbF associated variants (Supplementary Table S3**)**, we calculated a Genetic Modifier Score based on five major QTLs (Fig. 2). This analysis revealed a significant positive correlation between the score and HbF levels (Spearman *r* = 0.395, *p* = 0.002), although the linear regression model explained only ∼14% of the variance (R² = 0.14, *p* = 0.004).

## Discussion

Studies to increase gamma globin expression pharmacologically or by using various genome editing technologies continue to be a focus of current research [10–12]. In addition, various endogenous genomic variations found in individuals are associated with naturally high HbF. In this study, we aimed to investigate the associations between elevated HbF levels and other HbF-related variants in the *BCL11A*, *HMIP*, and *HBG2* loci as well as potential functional variants in the recently described modifier gene *LRF/ZBTB7A*.

The genetic variants in these three loci account for approximately 20–50% of the variation in HbF levels in patients with SCA and β-thalassemia, as well as in healthy adults [13].

Our univariate analysis suggested that the *XmnI-HBG2* polymorphism was associated with an increase in HbF level by approximately 7.0% in our cohort. Similarly, we showed that individuals with the T allele for *XmnI-HBG2* had higher HbF levels than the mean HbF level of 8.8 in the patients in our study. One study revealed that *XmnI-HBG2* contributes 10.2% of the HbF variance in the healthy European population, whereas SNPs in the *BCL11A* and *HMIP* loci are responsible for approximately 15.1% and 19.4%, respectively [14]. A study showing that the *XmnI-HBG2* polymorphism, but not SNPs of the *BCL11A* or *HMIP* loci, has HbF-increasing properties in the hematologically normal Iranian population [15], similar to our study, although we included patients with high HbF levels in our Turkiye cohort. One study included 100 patients with β-thalassemia and 100 patients with SCA; *XmnI-HBG2* polymorphisms, *BCL11A* (rs1427407) and *HMIP* (rs9399137, rs66650271), have been associated with both high HbF values and low disease severity [16].

*LRF/ZBTB7A* was identified as a second independent repressor of HbF (Fig S1), and subsequent studies quickly established the link between *KLF1* mutations and elevated HbF levels through the regulation of both *BCL11A* and *LRF/ZBTB7A* [17]. Mutations in the *LRF/ZBTB7A* gene are responsible for macrocephaly, neurodevelopmental delay, lymphoid hyperplasia, and persistent fetal hemoglobin [18] (MNDLFH, OMIM #619769). The patient presented an HbF level of 100% at 1 month of age (reference HbF level at this age: ~70%). At 6 years and 5 months, coincident with the diagnosis of MNDLFH, genetic analysis revealed a de novo missense variant in the *LRF/ZBTB7A* gene (p.Cys384Trp). In another study in which patients with MNDLFH were involved, the fraction of HbF was found to be elevated in four of the five individuals with *LRF/ZBTB7A* gene mutations [19]. The heterozygous variants detected in the cohort included p.Glu278* (associated HbF level: 4.4%), p.Thr405Lys (5.3%), p.Val417GlyfsTer123 (11.1%), and p.Arg530GlyfsTer27 (2.2%).

We detected a p.P241L change (Fig. 3a) in a patient with an HbF level of 3.5 (Table 2). Moreover, we detected a synonym variation (p.Asp344Asp, Fig. 3c) in a patient with high HbF (5.1%). However, we did not find these two variations in the control group (*n* = 60), but we detected a novel in-frame deletion (p.Glu277del, Fig. 3b) in the *LRF/ZBTB7A* gene in an adult female in our control group whose HbF level was 1.5%. Therefore, our study reports the presence of *LRF/ZBTB7A* gene variants in both patients with TDBT and in individuals from the control group (% HbF level ≤ 2). The allele frequency of c.722 C > T (p.Pro241Leu) was found to be approximately 4 times greater in the Turkiye population than in the gnomAD (0.19% vs. 0.05%) database. With respect to c.1032 C > T (p.Asp344=), the allele frequency in Turkiye is more than 2 times higher than that in gnomAD (0.31% vs. 0.14%). The synonymous variant p.Asp344Asp (rs112339723) was identified in a patient with high HbF (5.1%). In silico analyses (CADD score: 3.14; AlphaMissense: 0.0 ‘likely benign’) did not provide evidence for a deleterious impact or an effect on splicing or local translation efficiency. Therefore, while this correlative finding in a patient with notably high HbF is interesting, the functional relevance of this specific variant remains uncertain. Its effect may be contingent on the genetic context, as this individual was also homozygous for the known HbF-booster SNP rs10189857 in *BCL11A*. This case highlights the potential for combined effects of known QTLs with other rare or neutral-appearing variants, the significance of which may only become apparent in specific genetic backgrounds and warrant further investigation in larger cohorts. Moreover, the novel *LRF/ZBTB7A* in-frame deletion (rs1361497779) was identified in a healthy control individual with normal hematological parameters and low HbF (≤ 2%), suggesting its effect on HbF modulation may be context-dependent.

*LRF/ZBTB7A* gene expression levels in patients who received hydroxyurea treatment (homozygous SCA or combined heterozygous sickle cell/beta thalassemia) were found to be lower than those in patients who responded to treatment, as expected [20]. Notably, analysis of the K562 genomic data revealed no evidence of large deletional HPFH mutations, suggesting that its high HbF phenotype may involve alternative regulatory mechanisms such as the *LRF/ZBTB7A* variants investigated here. Persistent and high fetal hemoglobin levels were achieved in pediatric TDBT patients in whom *BCL11A* was disrupted by CRISPR-Cas9-mediated genome editing [21]. *BCL11A* is a promising candidate for treating β-hemoglobinopathies because of its high degree of HbF resurgence, lack of off-target effects, and unaltered gene expression, whereas the HBG1/2 approach also achieves clinically relevant HbF levels with a mediocre safety profile, suggesting that further research could lead to the development of viable alternative gene therapies for such diseases [22, 23]. Our findings are in line with one of the latest CRISPR/Cas9-based studies showing that disruption of either the *LRF/ZBTB7A* or *BCL11A* binding site comparably reactivates γ-globin expression, suggesting that both are viable therapeutic targets for β⁰-thalassemia [24]. This is further supported by recent pharmacological evidence demonstrating that targeted degradation of *LRF/ZBTB7A* robustly induces HbF without significant toxicity, reinforcing its therapeutic potential [25].

The significant yet modest explanatory power (R² ~14%) of the Genetic Modifier Score confirms the aggregate contribution of known QTLs to HbF variation, while also indicating that the majority of the phenotypic variance is driven by other factors. This unexplained variance provides a compelling genetic context for the putative role of novel modifiers, such as the *LRF/ZBTB7A* variants identified here, which may exert effects powerful enough to override the predisposition set by known QTLs.

The statistical power of our study was constrained by the cohort size, which may have been insufficient to detect associations for the other SNPs in *BCL11A* and *HBS1L-MYB*, especially given their previously reported modest effect sizes. Furthermore, it should be noted that our study was not designed to compare the modifier score between patients and controls, as the score itself is predicated on the presence of an elevated HbF phenotype. This limitation should be considered when interpreting the non-significant findings for these loci.

## Conclusions

In our study, the *XmnI-HBG2* polymorphism accounted for approximately 7% of the variance in fetal hemoglobin levels in transfusion-dependent beta thalassemia patients with high HbF levels. By examining the genomic and transcriptomic data of the K562 cell line, we identified certain variants in the *LRF/ZBTB7A* gene that we hypothesize may contribute to the variation in HbF levels. Our study demonstrates the utility of leveraging genomic data from model cell lines to generate testable hypotheses for clinical genetic modifiers. Nevertheless, the potential of cell lines to provide researchers with data for a variety of quantitative measurements must be considered and taken into account. Bioinformatics data from cell cultures can reveal pinpoint information about specific characteristics in disease groups that have not been previously investigated.

Our study reports novel, rare variants in the *LRF/ZBTB7A* gene, suggesting their potential as candidate genetic modifiers that may contribute to the variation in HbF levels among TDBT patients. While the correlative data are suggestive, definitive confirmation of their functional impact and therapeutic relevance awaits future studies using targeted in vitro models.

Collectively, the modest effect of the cumulative Genetic Modifier Score (R²~14%) and the identification of novel *LRF/ZBTB7A* variants suggest that a significant portion of HbF variability in our cohort arises from yet to be discovered genetic factors, with *LRF/ZBTB7A* emerging as a prime candidate for such a modifier.

The findings of this study should be interpreted within its limitations. The sample size, while valuable for a rare disease, limits the statistical power for robust subgroup analyses and broad generalizations. Furthermore, the genetic modifier score applied here is a hypothesis-generating model based on literature-derived SNPs, and its predictive value requires validation in larger, independent cohorts. It is also important to note that comprehensive clinical data (such as splenectomy status and *HBB* genotypes for the entire cohort) was not the primary focus of this genetic association study and was therefore only available and reported for the illustrative cases where a specific *LRF/ZBTB7A* mutation was found. Finally, the focus on specific SNPs and the lack of functional validation in experimental models mean that other significant genetic factors and the precise biological impact of the identified *LRF/ZBTB7A* variants may not be fully captured.

## Supplementary Information

Supplementary Material 1.

Supplementary Material 2.

Supplementary Material 3.

Supplementary Material 4.Figure S1. TPM-level expression of *LRF*/*ZBTB7A*, *BCL11A*, and *XmnI-HBG2* in K562 cells. K562 cells expressed*LRF*/*ZBTB7A* (2.8 ± 0.7 TPM), *BCL11A* (1.6 ± 1.6 TPM), and *HBG2* (0.7 ± 1.6 TPM) transcripts. *ACTB* (β-actin) is shown as a housekeeping gene for reference. Gene expression values (TPM: transcripts per kilobase million, mean ± SD) were retrieved from the DepMap portal (DepMap ID: ACH-000551; file: OmicsExpressionProteinCodingGenesTPMLogp2.csv) via https://depmap.org/portal/. The right panel shows that *LRF*/*ZBTB7A* and *BCL11A* independently repress *XmnI-HBG2* expression.

## Data Availability

All data generated or analysed during this study are included in this published article and its supplementary information files. The variant data generated during this study have been deposited in the ClinVar repository under the temporary submission handles SUB15634423, SUB15634458 and SUB15634616.The permanent accession numbers (SCV) for these variants are available and can be accessed via our submitter page on the NIH ClinVar database (https://www.ncbi.nlm.nih.gov/clinvar/submitters/510189).

## Abbreviations

ACMG: American College of Medical Genetics and Genomics
BCL11A: B-cell lymphoma/leukemia 11A (gene)
CRISPR: Clustered Regularly Interspaced Short Palindromic Repeats
HbF: Fetal hemoglobin
HBB: Hemoglobin subunit beta (gene)
HBG2: Hemoglobin subunit gamma 2 (gene)
HMIP: HBS1L-MYB intergenic polymorphism (locus)
LD: Linkage disequilibrium
LRF: Leukemia/lymphoma-related factor (same as *ZBTB7A*)
MNDLFH: Macrocephaly, neurodevelopmental delay, lymphoid hyperplasia, and persistent fetal hemoglobin (syndrome)
OMIM: Online Mendelian Inheritance in Man (database)
QTL: Quantitative trait locus/loci
RDBH: Reverse dot blot hybridization
TDBT: Transfusion-dependent beta thalassemia
VUS: Variant of uncertain significance
XmnI-HBG2: A specific polymorphism (rs7482144) in the *HBG2* gene
ZBTB7A: Zinc Finger and BTB Domain Containing 7A (gene)
