# Supplementary material for: Novel LRF/ZBTB7A variants and known HbF-modulating SNPs in transfusion-dependent β-thalassemia
Source: BMC Med Genomics. 2025 Dec 18;18:194. doi: 10.1186/s12920-025-02275-5 (PMC12713296; doi:10.1186/s12920-025-02275-5)
Supplement: Supplementary file 1 — Supplementary Material 1. [file 12920_2025_2275_MOESM1_ESM.docx]

**Supplementary Table 1**

**Table S1. XmnI-HBG2 explains 7.0% variation of total HbF level.**

| **Tests of Between-Subjects Effects*** | | | | | | |
| --- | --- | --- | --- | --- | --- | --- |
| Dependent Variable: HBF | | | | | | |
| Source | Type III Sum of Squares | df | Mean Square | F | Sig. | Partial Eta Squared |
| Corrected Model | 136,823^a^ | 2 | 68,412 | 2,286 | ,111 | ,078 |
| Intercept | 281,689 | 1 | 281,689 | 9,411 | ,003 | ,148 |
| Age | 14,405 | 1 | 14,405 | ,481 | ,491 | ,009 |
| XmnI_HBG2_T_Allele | 122,523 | 1 | 122,523 | 4,093 | ,048 | ,070 |
|  | 1616,367 | 54 | 29,933 |  |  |  |
| Total | 6016,170 | 57 |  |  |  |  |
| Corrected Total | 1753,190 | 56 |  |  |  |  |
| 1. R Squared = ,078 (Adjusted R Squared = ,044) *Univariate analysis | | | | | | |
