# Supplementary material for: Novel LRF/ZBTB7A variants and known HbF-modulating SNPs in transfusion-dependent β-thalassemia
Source: BMC Med Genomics. 2025 Dec 18;18:194. doi: 10.1186/s12920-025-02275-5 (PMC12713296; doi:10.1186/s12920-025-02275-5)
Supplement: Supplementary file 2 — Supplementary Material 2. [file 12920_2025_2275_MOESM2_ESM.docx]

**Table S2. Key hematological and genetic data of the study participants, including hemoglobin analysis and *HBB* genotypes.**

|  | **Control** | | **TDBT** | | | | |
| --- | --- | --- | --- | --- | --- | --- | --- |
| ID | HBF% | HBA2% | HBF % | HBA2 % | HGB (g/dL) | MCV (fL) | Genotype (*HBB* gene, OMIM *141900) |
| **1^+^** | **1.5** | **5.9** | 27.4 | 4 | 8.2 | 75.8 | -30T>A/-30T>A |
| 2 | 1.7 | 4 | 25 | 3 | 9.2 | 75.4 | IVS. I.110 (G>A)/IVS. I.110 (G>A) |
| 3 | 0.6 | 3.8 | 20.1 | 3.3 | 8.9 | 83.2 | IVS. I.110 (G>A)/IVS. I.110 (G>A) |
| 4 | 1.7 | 5 | 17.6 | 2.6 | 8.6 | 83.7 | IVS. I.110 (G>A)/IVS. I.110 (G>A) |
| 5 | 0.3 | 2.5 | 17 | 3 | 8.4 | 74.6 | IVS. I.110 (G>A)/IVS. I.110 (G>A) |
| 6 | 0.2 | 2.5 | 17 | 2.7 | 9.1 | 83.7 | IVS. I.110 (G>A)/IVS. I.110 (G>A) |
| 7 | 1.2 | 2.6 | 16.7 | 3.1 | 8.9 | 78.3 | IVS. I.1(G>A)/IVS. I.110 (G>A) |
| 8 | 0.4 | 2.3 | 14.3 | 3.2 | 8.7 | 85.2 | IVS. I.110 (G>A)/IVS. I.6 (T>C) |
| 9 | 0.4 | 3 | 14.1 | 2.8 | 8.9 | 86.9 | IVS. I.110 (G>A)/IVS. I.110 (G>A) |
| 10 | 0.9 | 2.6 | 13.6 | 3.4 | 10 | 75.4 | NA |
| 11 | 1.6 | 2.6 | 13.4 | 3.9 | 7.1 | 72.7 | (-30T>A/-30T>A) |
| 12 | 0.4 | 2.8 | 12.8 | 2.7 | 8.9 | 84.4 | IVS. I.110 (G>A)/IVS. I.110 (G>A) |
| 13 | 0.2 | 2.9 | 12.5 | 3.1 | 9.2 | 85.5 | IVS. I.110 (G>A)/IVS. I.110 (G>A) |
| 14 | 0.2 | 4.1 | 12.3 | 3 | 8.8 | 81.9 | IVS. I.110 (G>A)/IVS. I.110 (G>A) |
| 15 | 0.4 | 4 | 12.1 | 3 | 8.5 | 75 | IVS. I.110 (G>A)/IVS. I.110 (G>A) |
| 16 | 0.2 | 3 | 11.6 | 2.8 | 8.5 | 74.8 | NA |
| 17 | 0.2 | 4 | 11.1 | 2.8 | 8.3 | 84.2 | IVS. I.110 (G>A)/IVS. I.110 (G>A) |
| 18 | 0.3 | 3.8 | 11 | 2.6 | 8.6 | 75.7 | IVS. I.110 (G>A)/HBE |
| 19 | 1.2 | 2.9 | 10.8 | 2.6 | 9.3 | 78.3 | IVS. I.1 (G>A)/IVS. I.1 (G>A) |
| 20 | 0.3 | 3.9 | 10.5 | 2.8 | 10.2 | 82.5 | IVS. I.110 (G>A)/IVS. I.110 (G>A) |
| 21 | 0.3 | 2.8 | 16.7 | 2.8 | 7.8 | 77.3 | IVS. II.1 (G>A)/IVS. II.745 (C>G) |
| 22 | 1.4 | 4.2 | 9.8 | 2.7 | 7.8 | 77.8 | IVS. I.110 (G>A)/IVS. I.110 (G>A) |
| 23 | 1.1 | 3.4 | 8.8 | 3.1 | 9.5 | 74 | IVS. I.110 (G>A)/IVS. I.6 (T>C) |
| 24 | 0.2 | 3.1 | 5.8 | 3 | 8.9 | 82.5 | NA |
| 25 | 0.3 | 1.5 | 8.6 | 2.5 | 10 | 79.4 | IVS. I.110 (G>A)/IVS. II.1 (G>A) |
| 26 | 0.3 | 2.8 | 8.47 | 5.5 | 6.7 | 73.1 | IVS. I.110 (G>A)/IVS. I.110 (G>A) |
| 27 | 1.7 | 4 | 8.3 | 2.5 | 9.3 | 81.5 | IVS. I.1 (G>A)/IVS. I.110 (G>A) |
| 28 | 0.6 | 3.8 | 8.2 | 2.6 | 9.2 | 77.8 | IVS. I.1 (G>A)/IVS. I.1 (G>A) |
| 29 | 1.7 | 5 | 8 | 2.9 | 8.4 | 81.7 | IVS. I.6 (T>C)/IVS. I.110 (G>A) |
| 30 | 0.3 | 2.5 | 7.5 | 3.7 | 8.9 | 71.1 | IVS. I.6 (T>C)/IVS. I.6 (T>C) |
| 31 | 0.2 | 2.5 | 7.5 | 4.4 | 7.5 | 74.5 | IVS. I.6 (T>C)/IVS. I.6 (T>C) |
| 32 | 1.2 | 2.6 | 7.2 | 6 | 6.5 | 55.1 | NA |
| 33 | 0.2 | 3.1 | 7 | 5.6 | 8.8 | 71.8 | IVS. I.110 (G>A)/IVS. I.110 (G>A) |
| 34 | 0.4 | 2.9 | 6.1 | 2.8 | 10 | 85.8 | IVS. I.110 (G>A)/IVS. I.110 (G>A) |
| 35 | 0.2 | 2.4 | 6 | 2.7 | 10.1 | 84.2 | IVS. I.110 (G>A)/IVS. I.110 (G>A) |
| 36 | 0.7 | 2.9 | 5.9 | 3.9 | 9.2 | 78.6 | IVS. I.6 (T>C)/IVS. I.6 (T>C) |
| 37 | 0.6 | 2.7 | 5.8 | 2.8 | 8.5 | 79.8 | IVS. I.110 (G>A)/IVS. I.110 (G>A) |
| 38 | 0.6 | 2.9 | 5.6 | 2.8 | 10.6 | 83.2 | IVS. I.110 (G>A)/IVS. I.110 (G>A) |
| **39^&^** | 0.4 | 2.8 | **5.1** | 2.4 | 8.2 | 77.9 | **IVS. I.110 (G>A)/IVS. I.110 (G>A)** |
| 40 | 0.2 | 2.7 | 5.1 | 2.9 | 8.1 | 84.8 | IVS. I.110 (G>A)/IVS I.110 (G>A) |
| 41 | 0.4 | 2.6 | 4.7 | 3.3 | 8.6 | 89.3 | IVS. I.6 (T>C)/IVS. I.110 (G>A) |
| 42 | 0.4 | 2.3 | 4.7 | 2.7 | 9.6 | 81.1 | IVS. I.110 (G>A)/IVS. I.110 (G>A) |
| 43 | 2 | 2.5 | 4.6 | 2.9 | 7.4 | 84.4 | IVS. I.110 (G>A)/IVS. I.110 (G>A) |
| 44 | 1.9 | 2.7 | 4.5 | 2.9 | 9.1 | 79.7 | IVS. I.110 (G>A)/IVS. I.110 (G>A) |
| 45 | 1.7 | 2.2 | 3.8 | 3.2 | 10 | 85.2 | IVS. I.110 (G>A)/IVS. I.110 (G>A) |
| 46 | 0.6 | 2.6 | 3.8 | 4 | 6.4 | 80 | IVS. I.6 (T>C)/IVS. I.6 (T>C) |
| 47 | 0.9 | 2.6 | 3.8 | 2.8 | 9.5 | 80.8 | IVS. I.110 (G>A)/IVS. I.110 (G>A) |
| 48 | 1.6 | 2.6 | 3.6 | 2.6 | 9.9 | 79.4 | IVS. I.110 (G>A)/IVS. I.110 (G>A) |
| 49 | 0.3 | 2.7 | 3.5 | 10 | 8.2 | 45.8 | NA |
| **50^#^** | 1.90 | 2.8 | **3.5** | 2.8 | 9.5 | 85.4 | **-30T>A/IVS. I.110 (G>A)** |
| 51 | 1.6 | 3.5 | 2.8 | 2.7 | 8.1 | 79.5 | IVS. I.110 (G>A)/IVS. I.110 (G>A) |
| 52 | 1.50 | 2. ^&^6 | 2.8 | 2.8 | 8.3 | 79.9 | IVS. I.6 (T>C)/IVS. I.6 (T>C) |
| 53 | 1 | 5 | 2.7 | 2.7 | 9.4 | 85.5 | IVS. I.110 (G>A)/IVS. I.110 (G>A) |
| 54 | 0.4 | 2.6 | 2.7 | 2.9 | 9.6 | 83 | IVS. I.110 (G>A)/IVS. I.110 (G>A) |
| 55 | 1.7 | 2.2 | 2.6 | 2.5 | 8.7 | 76.3 | IVS. I.110 (G>A)/IVS. I.110 (G>A) |
| 56 | 1.3 | 2.7 | 2.3 | 2.6 | 9.2 | 77.6 | IVS. I.110 (G>A)/IVS I.110 (G>A) |
| 57 | 1.50 | 2.4 | 2.2 | 2.5 | 9.2 | 78.1 | IVS. I.110 (G>A)/IVS. II.745(C>G) |
| 58 | 0.6 | 4.1 | 2.2 | 2.6 | 9.5 | 85.1 | IVS. I.110 (G>A)/IVS. I.110 (G>A) |
| 59 | 2 | 2.5 |  |  |  |  |  |
| 60 | 1.4 | 2.9 |  |  |  |  |  |

^+^Number 1 is the case with p.E277del variation. ^&^Number 39 is the patient with TDBT and p.D344D variation. ^#^Number 50 is the patient with TDBT and p.P251L mutation. NA: Data is not available. 60 controls are not directed to *HBB* genotyping since no clinical indication is present. TDBT patients are diagnosed and followed by the clinicans by routine procedures.
