# Supplementary material for: Novel LRF/ZBTB7A variants and known HbF-modulating SNPs in transfusion-dependent β-thalassemia
Source: BMC Med Genomics. 2025 Dec 18;18:194. doi: 10.1186/s12920-025-02275-5 (PMC12713296; doi:10.1186/s12920-025-02275-5)
Supplement: Supplementary file 3 — Supplementary Material 3. [file 12920_2025_2275_MOESM3_ESM.docx]

**Table S3. Scoring system for the five HbF-associated SNPs used to calculate the Genetic Modifier Score.**

| **SNP** | **Zygosity** | **Allele** | **Genetic Modifier Score** |
| --- | --- | --- | --- |
| rs1427407 (G>T) | 0 | GG | 0 |
|  | 1 | GT | 1 |
|  | 2 | **TT** | **2** |
| rs10189857 (A>G) | 0 | **AA** | **2** |
|  | 1 | AG | 1 |
|  | 2 | GG | 0 |
| rs28384513 (A>C) | 0 | **AA** | **2** |
|  | 1 | AC | 1 |
|  | 2 | CC | 0 |
| rs9399137 (T>C) | 0 | TT | 0 |
|  | 1 | TC | 1 |
|  | 2 | **CC** | **2** |
| rs7482144 (C>T) | 0 | CC | 0 |
|  | 1 | CT | 1 |
|  | 2 | **TT** | **2** |

The total score is the sum of points assigned to each genotype, with a theoretical maximum of 10 points. For example, a patient with a genotype code of "2-1-0-0-0" would receive a total of 3 points (calculated as 2 + 1 + 0 + 0 + 0). This code is interpreted as: 2 points (homozygous) for rs1427407, 1 point (heterozygous) for rs10189857, and 0 points (homozygous wild-type) for rs28384513, rs9399137, and rs7482144
