# Supplementary figures and images for: Novel LRF/ZBTB7A variants and known HbF-modulating SNPs in transfusion-dependent β-thalassemia
Source: BMC Med Genomics. 2025 Dec 18;18:194. doi: 10.1186/s12920-025-02275-5 (PMC12713296; doi:10.1186/s12920-025-02275-5)

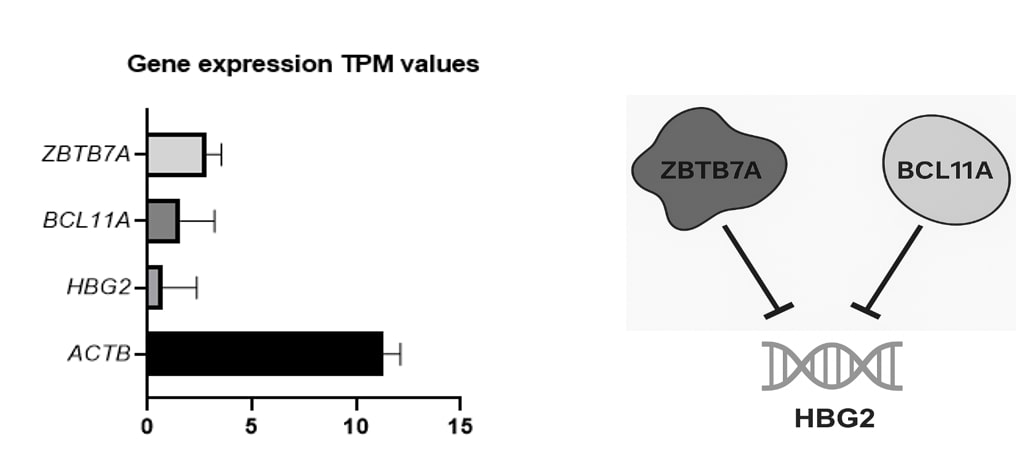

Supplement: Supplementary file 4 — Supplementary Material 4.Figure S1. TPM-level expression of LRF/ZBTB7A, BCL11A, and XmnI-HBG2 in K562 cells. K562 cells expressedLRF/ZBTB7A (2.8 ± 0.7 TPM), BCL11A (1.6 ± 1.6 TPM), and HBG2 (0.7 ± 1.6 TPM) transcripts. ACTB (β-actin) is shown as a housekeeping gene for reference. Gene expression values (TPM: transcripts per kilobase million, mean ± SD) were retrieved from the DepMap portal (DepMap ID: ACH-000551; file: OmicsExpressionProteinCodingGenesTPMLogp2.csv) via https://depmap.org/portal/. The right panel shows that LRF/ZBTB7A and BCL11A independently repress XmnI-HBG2 expression. [file 12920_2025_2275_MOESM4_ESM.jpg]
